# Assessing Program Culture in Virtual Fellowship Interviews: Insights From Pulmonary and Critical Care Fellows

**DOI:** 10.7759/cureus.77466

**Published:** 2025-01-15

**Authors:** Geneva Tatem, Eric Snowden, Andrea Williams, Mara M Hoffert, Karla D Passalacqua

**Affiliations:** 1 Pulmonary and Critical Care Medicine, Henry Ford Health System, Detroit, USA; 2 Pulmonary and Critical Care Medicine, AdventHealth Medical Group, Merriam, USA; 3 Graduate Medical Education, Henry Ford Health System, Detroit, USA

**Keywords:** fellowship, interview, organizational culture, qualitative research, recruitment activity

## Abstract

Background: Virtual interviewing for fellowship training programs has been widely adopted since the COVID-19 pandemic. However, whether fellowship candidates can adequately evaluate training program culture through virtual interviews is unclear.

Objective: Our aim was to explore how pulmonary and critical care fellows ascertained program culture during virtual and in-person fellowship program recruitment interviews, with the overall goal of improving our virtual recruiting interview processes.

Methods: Exploratory semi-structured one-on-one interviews (study-interviews) following a constructivist approach were done during the fall of 2022 with a convenience sample of current fellows within the pulmonary critical care medicine fellowship program in an urban tertiary care hospital. Questions probed fellows’ perspectives on program culture, what features of program culture they valued, and how they evaluated program culture during their initial fellowship interviews (recruitment-interviews). Study-interviews were framed to explore four deductive themes, and transcripts were analyzed with inductive thematic analysis.

Results: Of the 11 fellows interviewed, two had completed in-person and nine had completed virtual recruitment-interviews. There was an overall favorable perception of program culture during all recruitment-interviews, regardless of format. Elements of program culture that fellows valued included training program quality, an academic focus, complexity in cases, workplace diversity, a positive socioemotional environment, and a collaborative/supportive working/learning environment.

Conclusions: This study suggests that important elements of program culture can be evaluated by fellowship candidates through virtual interviews when applicants are allowed ample opportunity for high-quality interactions with faculty and current trainees.

## Introduction

The COVID-19 pandemic necessitated many changes in medical education, most prominently a need for distance learning due to physical distancing requirements [1]. As a result, residency and fellowship training programs adopted virtual online interviewing as a necessary means to recruit candidates during the pandemic [2,3]. However, not being physically present at an institution during the interview process could make evaluating a program of interest challenging for applicants. Importantly, now that the pandemic has ended, fellowship training programs continue to explore the benefits and drawbacks of in-person versus virtual interviews [2,3].

The need for virtual interviewing during the pandemic revealed that virtual interviews have several advantages relative to in-person interviews, including being trainee-centric, equitable, fiscally responsible, and environmentally advantageous [1-4]; therefore, some professional societies support continuing the use of virtual interviews [1-4]. However, certain elements of the interviewing process could be difficult through a virtual medium, such as evaluating program culture. Culture may be particularly difficult to assess virtually because the online format limits the ability of participants to view group dynamics, sense non-verbal cues, and physically experience the entire institutional environment. Some research has shown that trainees sense these limitations. For example, a study of pulmonary and critical care medicine (PCCM) fellowship candidates found that most applicants preferred an in-person component to complement virtual interviews, and most participants believed that virtual interviews hindered their ability to evaluate training program culture [5]. Additionally, in a survey of residency candidates in the 2021 National Resident Matching Program, nearly half of the respondents felt that determining program culture from web-based materials and virtual interviews was very challenging [6]. But given that virtual interviewing is likely here to stay, fellowship program directors need to determine whether candidates can get a good sense of a program's culture through virtual interviews and if strategies can be developed to help virtual interviewees assess program culture. Thus, determining how virtual interviews help or hinder fellowship applicants in evaluating program culture is needed to determine the best recruitment practices.

Therefore, using a phenomenological-constructivist paradigm, we performed an interview study to explore how our PCCM program fellows were able to evaluate program culture during their initial recruitment interviews. Because our group of fellows included those who had been recruited both before and during the COVID-19 pandemic, we sought to gather the perspectives of those who had each type of interview: in-person and virtual. Through semi-structured one-on-one interviews, we assessed fellows’ perspectives, values, expectations, and activities surrounding their initial fellowship recruitment interviews. Because program culture is a shared, communal feature of medical training, our qualitative approach situates our study within the communities of practice framework, which posits that communities establish social environments that support learning through relationships and interactions with others [7]. Our aims were to understand how applicants evaluated program culture during their recruitment interviews, how program alignment was perceived, and what aspects of program culture were most valued-with a particular emphasis on exploring the experiences of those fellows who participated in virtual interviews to better understand this approach. Understanding fellows’ unique experiences may reveal aspects of the interview process, both in-person and virtual, that can help graduate medical education (GME) programs accurately and honestly convey program culture to optimize medical training program recruitment.

## Materials and methods

Study setting and participants

This exploratory qualitative interview study was conducted between August and October 2022. Participants comprised a convenience sample of fellows in the Accreditation Council for GME (ACGME)-accredited PCCM fellowship program at a large urban academic healthcare institution in the state of Michigan. Semi-structured, one-on-one interviews based on a phenomenological-constructivist paradigm were performed to explore how the candidates constructed the meaning of training program culture and how they experienced program culture during their recruitment interviews. In the summer of 2022, all current fellows were invited by the program coordinator through email to participate. First-year and second-year fellows had done their recruitment interviews virtually in 2020 and 2021, whereas third-year fellows had participated in the last in-person interviews performed before pandemic restrictions in 2019. The program director was not provided participant names. No incentives were provided for participation. This study was evaluated and approved by the Henry Ford Health Institutional Review Board (approval #15801).

Data collection

For this study, the term “recruitment-interview” refers to the initial virtual or in-person interview fellows underwent during the PCCM training program recruitment process, while “study-interview” refers to the semi-structured interviews performed to explore the fellows’ experiences during their recruitment-interviews. The GME instructional design team (IDT) developed an 11-question semi-structured study-interview guide based on the study question and goals, and the final interview guide was selected by consensus (Table 1). Questions were designed to elicit information on how fellows evaluated PCCM program culture during recruitment-interviews and the aspects of program culture that fellows valued. Deductive themes were developed to frame the interview guide through the lens of the communities of practice framework, which emphasizes that learning is a social process occurring within the context of a community of individuals who share common interests and practices [8]. The following deductive themes were generated to elicit targeted information from fellows: (1) definitions of culture, (2) important valued aspects of program culture, (3) evaluation of program culture outside the interview process, and (4) evaluation of program culture during the interview process.

**Table 1:** Reflective interview guide PCCM: pulmonary and critical care medicine

Part 1: Background informational questions
What year of fellowship training are you in?
If you are willing, will you please share your gender identity? (man/woman/transgender man/transgender woman/prefer not to answer)
Did you train at Henry Ford Health as a resident?
Part 2: Professional culture definition
Interviewer states definition of culture: “Professional (organizational) cultures is the set of shared attitudes, values, goals, and practices that characterizes an institution or organization” [9].
What is the most important to you when considering a training program’s culture?
What information did you use to assess our culture during your interview process?
Probing: What was your impression of our culture based on your interview?
Interviewer states program culture and values: “Our program values excellence in clinical care for a diverse patient population and maintaining an environment of inclusion for patients, trainees, and faculty. We aim to do this by offering broad exposure to complex specialty care in an environment that is conducive to learning and professional growth.”
On a scale of 1 through 5, with 1 being “not clear at all” and 5 being “extremely clear,” how well did we portray our professional culture values during your interview?
Can you give an example of how we portrayed this?
How well do we do this? Were you able to tell this based on your interview?
Part 3: Program “fit”
What does “fit” within a training program mean to you?
How well do you feel you “fit” into the program?

One-hour study-interviews with fellows were conducted through Webex (Cisco Systems, Milpitas, CA, US) between August 2022 and October 2022. Operational definitions of general professional culture, the PCCM program culture, and details about our program values reflecting diversity and inclusion/belonging and fostering growth and learning were provided during the study-interviews (Table 1). Participants underwent an informed consent process in which they were informed that they could decline to answer any questions, their participation was voluntary, the interviews were being recorded, and their anonymity would be maintained. Study interviews were conducted by two different GME IDT members and recorded with Webex transcription software. Study interview transcripts were reviewed by researchers to remove all identifying information and confirm accuracy before analysis. Pilot testing of the interview guide was done by conducting two test interviews with members of the GME IDT to gauge technology effectiveness, the time required to perform a complete study interview, and the likely structure of responses. Responses from the pilot test interviews were compared to ensure that interviewees understood the questions similarly. Pilot interviews were not included in the final analysis.

Data analysis

Transcript coding and analysis were performed by two researchers who manually reviewed the interview transcripts and identified inductive themes and sub-themes that emerged during interviews within the framework of the aforementioned a priori deductive themes. No analysis software was used; manual analysis was preferred due to researcher expertise, the scope of the project, and the aim of seeking nuance and depth. Researchers were briefed on challenging their preconceptions and being open-minded and reflective, as well as avoiding personal biases and not imposing their own beliefs. Briefly, thematic analysis was performed in six steps: reviewers read through all transcripts to get an overall sense of responses; a priori themes from the Interview Guide were identified; reviewers discussed common emergent ideas and identification of a priori themes across transcripts, which were assigned descriptive codes; reviewers read the transcripts again highlighting quotations that matched a priori and emergent codes; reviewers discussed each transcript and how the coding was done until consensus was reached; finally, once consensus through discussion on discrepancies was reached and redundant ideas were combined, passages were recoded under final a priori and emergent codes. A total dataset for the thematic analysis was compiled consisting of 149 statements and ideas under the four deductive themes. Regarding researcher characteristics and reflexivity, both members of the analysis team were professional non-physician educators in the GME IDT with experience in designing and conducting educational programs in virtual and in-person settings. Careful consideration was given to how the researchers’ experiences in these settings shaped and informed their consideration of respondents’ experiences during recruitment-interviews. Additionally, both GME researchers have been involved in developing educational programs to promote diversity, equity, inclusion, and justice within medical education; therefore, researchers were careful not to impose their own beliefs regarding the system’s culture and inclusivity when evaluating respondents’ statements.

## Results

Participants

A total of 11 out of 21 PCCM fellows (52%) participated in this study. About half of the participants were first-year fellows (n = 6), and about half were women (n = 6). Most participating fellows (n = 9) did their residency training at another hospital. Of the 11 fellows, nine had participated in virtual recruitment-interviews (six first-year and three second-year fellows) and two third-year fellows did in-person recruitment-interviews (Table 2).

**Table 2:** Characteristics of pulmonary and critical care medicine fellows *First-year and second-year fellows had virtual initial interviews; third-year fellows had in-person initial interviews **See Table 1. Fellows rated the portrayal of culture at the initial fellowship interview with a 1 through 5 scale: 1 = not at all clear; 5 = extremely clear

Characteristic	N = 11
Fellowship year*, n (%)	
First year	6 (55)
Second year	3 (27)
Third year	2 (18)
Gender identity, n (%)	
Man	5 (45)
Woman	6 (55)
Did residency at institution, n (%)	2 (18)
Rating of the portrayal of culture during interview**, mean (SD), all fellows (n = 11)	4.6 (0.5)
Rating of the portrayal of culture during interview**, mean (SD), first year & second year only (virtual interviews, n = 9)	4.7 (0.5)

Themes 1 and 2: culture, fit, and valued aspects of program culture

Fellows were queried about their feelings and values surrounding professional culture and fit within a potential training program. Fellows most frequently defined culture as sharing common beliefs/values (n = 7) and collaboration with the team (n = 5) (Tables 3, 4). Fellows defined fit as being based on common beliefs and values. A third-year fellow said, “Fit means even if I’m not exactly the same as the people around me I can still find common ground.”

**Table 3:** Thematic analysis of fellows' values, expectations, and activities surrounding training and program culture

Deductive themes and inductive themes and sub-themes	Number of times mentioned
Theme 1: definitions of culture and fit	
Desired diversity	1
Common beliefs and values	10
Ability to communicate	1
Ability to collaborate	5
Theme 2: important valued aspects of program culture	
Socioemotional expectations and environment	17
Academic expectations	5
Diversity expectations	4
Theme 3: evaluation of program culture outside of the interview process	
Website	2
Information from others at institution	6
Prior training at institution	3
Theme 4: evaluation of program culture during the interview process	
Sub-theme: training program quality	
Academic/research focus	16
Diverse specialties	5
Complexity of cases	4
Sub-theme: diversity	
Diverse co-fellows	7
Diverse faculty/staff	7
Diverse patients	4
Sub-theme: socioemotional environment	
Leadership/faculty approachable	10
Feeling seen/heard	9
Personal support	6
Personal connections	9
Shared values	4
Sub-theme: working and learning environment	
Work collaboration	8
Work support	6

**Table 4:** Key quotations describing fellows' values, expectations, and activities around training program culture

Themes 1 and 2: definitions of culture and fit and important valued aspects
I think one of the most important things that I look at, when I’m looking at a training program’s culture is the respect between the trainees and the staff. So, are the trainees treated in a respectful manner? Their opinions are valued, and the staff members are treating them with respect. The same goes for training, treating their staff members with respect as well and the respect of within the institution for support staff as well. How are we interacting with nursing staff, respiratory therapist, and other support staff? Is that in a very respectful, cordial manner? [First-year fellow]
…and the last one, which actually became the deciding factor for a lot of things, was diversity. So, I would go on every program’s website and if I could not find people that looked like me, I wasn’t interested. [Third-year fellow]
Sub-theme 1: training program quality
It was very professional and very dedicated to the training and the education of the fellows. It seems like that for most of their priorities, was to make sure that the fellows were getting trained the best that they possibly could… [Second-year fellow]
Sub-theme 2: diversity
…the attendings that I interviewed with were, I could see the diversity in gender, I could see the diversity in ethnicities, even within the faculty itself, and the fellows that I met that day, which was important for me to see that. [Third-year fellow]
I think the most overt for me was the strong female presence in the program, which is not our national average for pulmonary critical care, but the fact that it’s a very male-dominant subspecialty and the fact that this program in particular has such heavy female presence… [Third-year fellow]
Sub-theme 3: socioemotional environment
I felt like even during the interview, [X] made me feel like this interview is all about you. I have read so much about you, I know every detail about you, and I want to know more about that. And I want you to feel that I genuinely want you to be in [the] program. And from the get go, when [X] spoke to me I felt like, whoa, like this [person] knows me. So, I feel like, you could see right from there that you are a valued member of this team. I have made that whatever extra effort I did in the background without even having met you. I’ve taken the time to know about you so that I can have a conversation with you about what you feel is important to you in a training program, and we don’t necessarily want you to come here just because we feel like you have really good grades, but I feel like as a person as a physician and a person, you have something that you can contribute, and we value your contribution. [Second-year fellow]
But even the questions aside, the conversation that we had around it [the fellowship interview process] felt very personable, and that was probably one of the biggest reasons I ranked [them] as number one, was because it was the most personable interview that I had, where I felt like you weren’t just trying to get down to business. You’re trying to figure out who I was. [Third-year fellow]
We had to be our true self while doing this interview process, because we didn’t know what the questions were. They were essentially clinical scenarios or situations, and when you answer those questions, it’s you who’s answering it, you’re not prepared to…you’ve not memorized answers. [Third-year fellow]
Sub-theme 4: working and learning environment
…the general culture of the fellowship is very, very helpful to anyone that reaches out for our help. So, if you have a consult for our service, or you want to send somebody to the ICU, they never scoff at you or they’re never mean or anything like that. [Second-year fellow]
…a real team approach. People were supported and they had resources and were able to kind of lean on each other and were able to improve based on feedback. [First-year fellow]

When asked what was most important when considering a training program’s culture, most respondents (n = 10) mentioned socioemotional factors, such as strong connections with others and mutual respect. A first-year fellow stated about the program, “It also was a program that valued me as an individual and what I brought to the table.”

Fellows also commented on expectations surrounding the academic aspects of the program as well as the program’s diversity (Tables 3, 4). A first-year fellow shared that they were “very open with learning from the fellows as well, and a training program that was committed to supporting me in my career aspirations. They had resources available for fellows to do research…or focus on an area of interest that will allow them to take their career to the next level.”

Theme 3: evaluation of program culture outside the interview process

Fellows were asked about information they used to evaluate the program’s culture during the recruitment-interview process. Fellows reported that they obtained information from others within the institution and training program, and two fellows had done previous training at the institution. Only two individuals mentioned the program website as an important source of information. One first-year fellow explained that they “used information from the website. I…looked at the list of the faculty members…I also looked at the list of fellows by year. I pretty much wanted to see where they trained, where they came from, and I also watched a few videos on the website to sort of get a sense of what the training program would be like.”

Theme 4: evaluation of program culture during the interview process

We asked fellows to numerically rate how well we portrayed our professional culture and values during their initial recruitment interviews (1 = not at all clear through 5 = extremely clear). Fellows responded with a mean score of 4.6, indicating that they felt the culture was clearly communicated during recruitment interviews (Table 2). Responses to questions regarding how well the PCCM program conveyed culture and how fellows were able to evaluate program culture during their recruitment interviews revealed the following four sub-themes: training program quality, diversity, socioemotional environment, and working environment (Tables 3, 4).

Sub-theme 1: Training Program Quality

**Figure 1:**
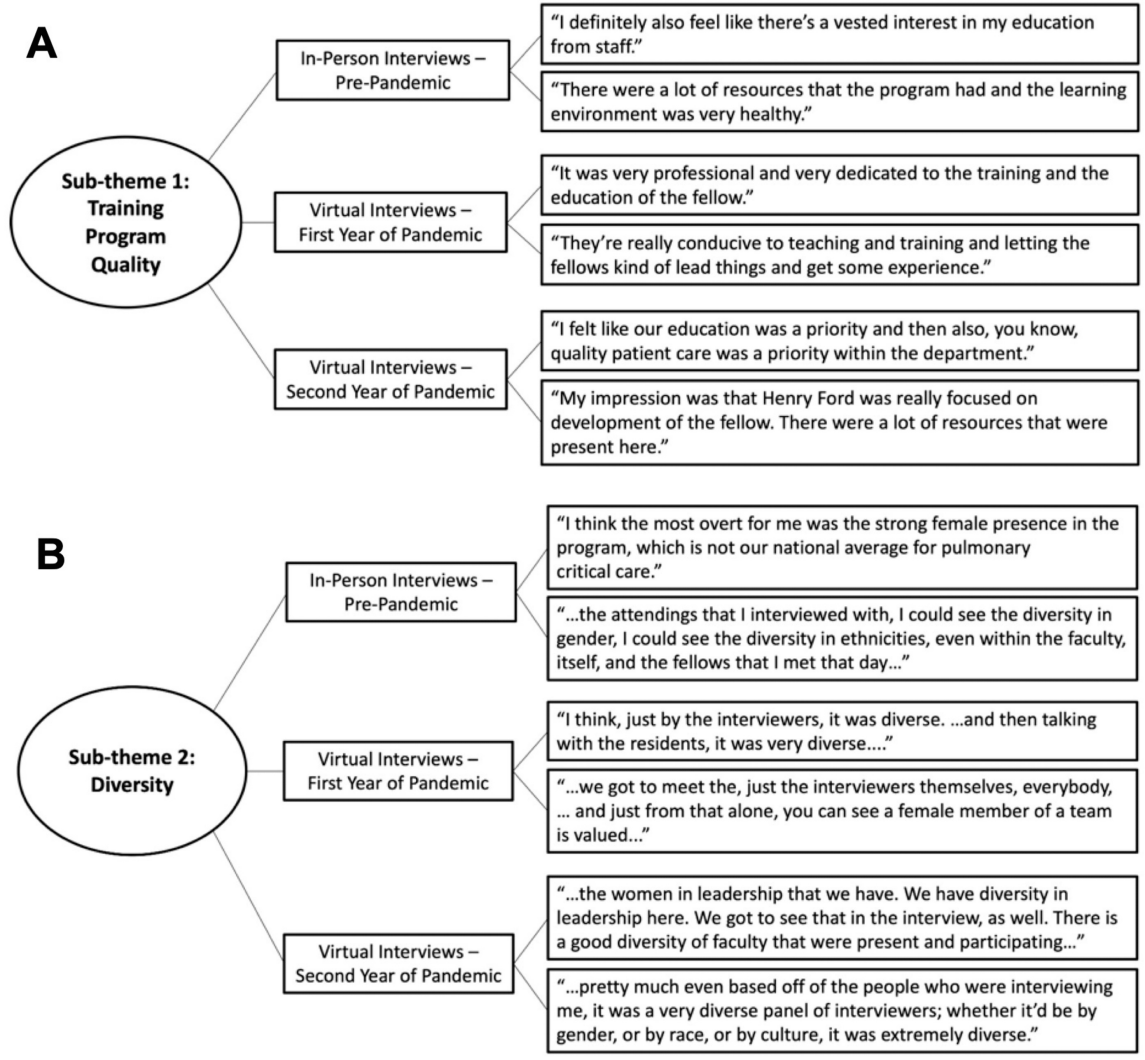
Select quotations describing fellows' perspectives on quality and diversity in training programs. Select quotations from interviews with fellows describing how they evaluated culture in the pulmonary and critical care medicine training program during their initial fellowship program recruitment-interviews. Quotations reflect two key sub-themes identified by thematic analysis: (A) training program quality and (B) diversity. Third-year fellows participated in in-person initial interviews in 2019. First-year and second-year fellows participated in virtual initial interviews due to COVID-19-related policies in 2021 and 2020, respectively

Of the 11 fellows, 10 indicated that academics and a research focus were priorities in evaluating training program quality. Diverse specialties and the complexity of medical cases were also mentioned as important aspects of program quality. Overall, fellows felt that everyone they spoke to had a genuine interest in the education and success of trainees (Figure 1A and Table 4). A first-year fellow said their impression during the virtual interview “was that (they were) really focused on development of the fellow. There was (sic) a lot of resources that are present here. And really, the focus is, what can this institution do to individually develop you and make you better? And that was demonstrated through the type of questions that I was asked about what my personal goals were for my career and really showing personal interest in what I wanted to do moving forward.”

Sub-theme 2: Diversity

Ten fellows indicated that diversity in faculty and co-fellows was important and they valued seeing their cultural representation in the program. Key quotations indicated that fellows appreciated the diversity and number of women in the program, both for faculty and current trainees (Figure 1B and Table 4). A first-year fellow mentioned that “…even based off the people who were interviewing me, it was a very diverse panel of interviewees; rather it’d be by gender, or by race, or by culture, it was extremely diverse. So, pretty much I got a sense that it wasn’t something that was talked about, but it was something that pretty much was portrayed and pretty much it was true.”

Sub-theme 3: Socioemotional Environment

**Figure 2:**
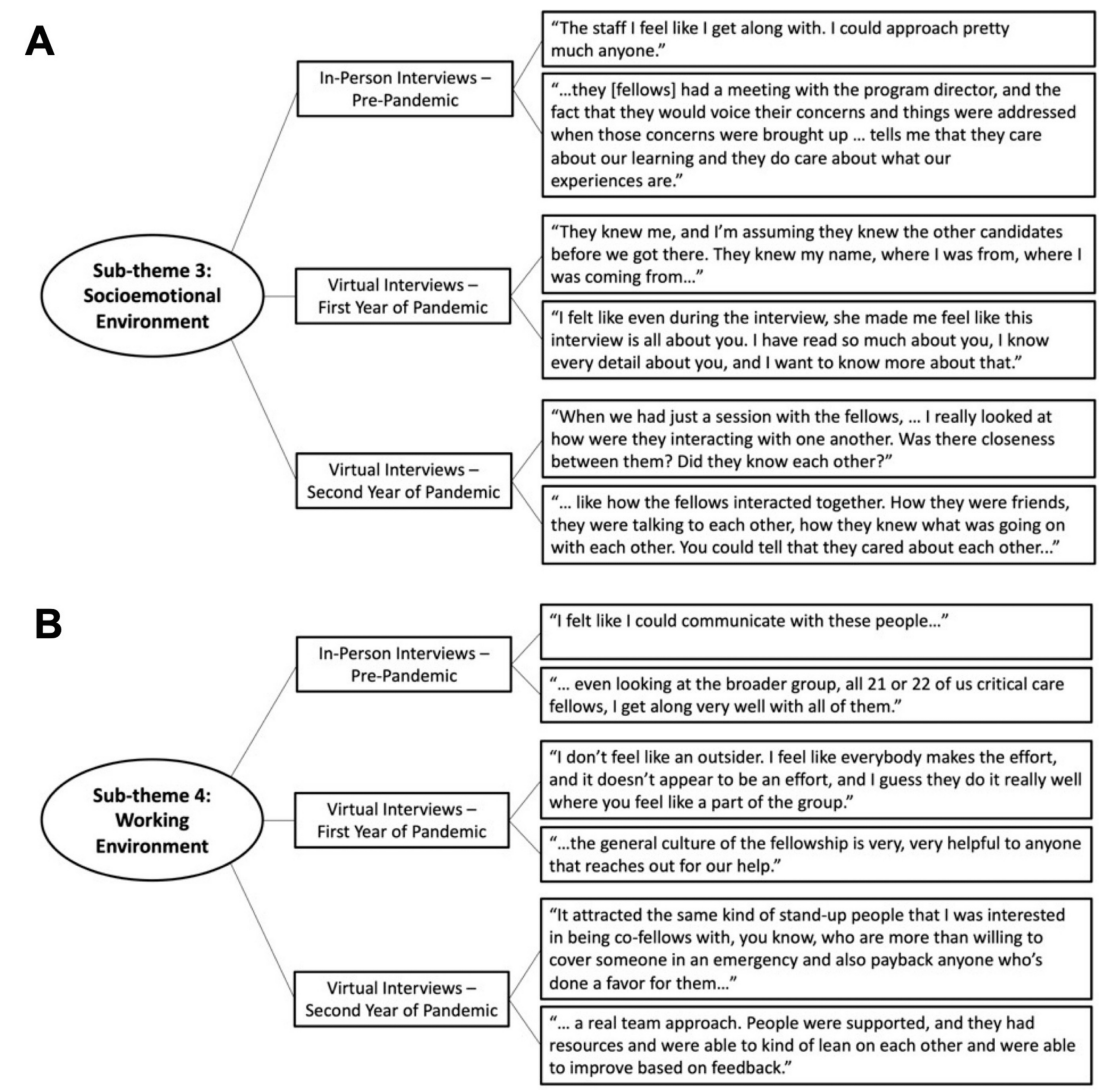
Select quotations describing fellows' perspectives on the socioemotional and working environments in training programs. Select quotations from interviews with fellows describing how they evaluated culture in the pulmonary and critical care medicine training program during their initial fellowship program recruitment-interviews. Quotations reflect two key sub-themes identified by thematic analysis: (A) socioemotional environment and (B) working environment. Third-year fellows participated in in-person initial interviews in 2019. First-year and second-year fellows participated in virtual initial interviews due to COVID-19-related policies in 2021 and 2020, respectively

There were 10 fellows who indicated personal and emotional aspects of program culture that they valued and had observed on their recruitment-interview days. Fellows felt that having leadership and faculty who were approachable and being seen and listened to were important. Additionally, the ability to make personal connections with others in the program was an important work environment feature (Figure 2A and Table 4). A first-year fellow explained that they “used the interaction between the fellows. When we had just a session with the fellows…I really looked at how were they were interacting with one another? Was there closeness in between them? Did they know each other? …to really see what the culture was like.”

Sub-theme 4: Working and Learning Environment

Having a collaborative and supportive work environment was an important aspect of culture for seven fellows. Responses suggested that fellows valued a sense of belonging, getting along with each other, helpfulness, and mutual support (Figure 2B and Table 4). One first-year fellow said, “It attracted the same kind of stand-up people that I was interested in being co-fellows with. You know, who are more than willing to cover someone in an emergency and also pay back anyone who’s done a favor for them kind of thing…always available for a question or a consult or concern.”

## Discussion

In this exploratory interview study, fellows in a PCCM program shared their perspectives on how they evaluated training program culture during their initial fellowship recruitment interviews and revealed that even when interviews were done through a virtual format, the fellows were able to identify the features of program culture that were important to them. Elements of program culture such as training program quality, workplace diversity, a positive socioemotional environment, and a collaborative, supportive working/learning environment were key priorities.

Medical training program culture is a critical element for applicants to consider when seeking a residency or fellowship. While the overall benefits and drawbacks of virtual interviewing have been discussed [10-13], determining whether culture can be adequately evaluated through virtual interviews is unclear. A survey study of 287 Family Medicine program directors showed that while 72.9% of respondents felt that in-person interviews would provide applicants with a comprehensive sense of their alignment with the program’s culture, values, and mission, 82.4% said that they were able to communicate values through a virtual format [14]. Additionally, a study of 565 residency and clinical psychology program interviewees revealed that 62% felt they were able to learn about program culture virtually [15]. Several explanations have been put forward regarding the challenges of conveying culture through a virtual format. One idea is that culture is an “intangible” aspect of the interview experience [16]. Also, virtual interviews do not provide opportunities for spontaneous conversations and may limit the number of personal interactions for interviewees [13]. Our applicants, however, felt that our program culture was portrayed clearly during virtual interviews, and we propose that more rigorous processes for specifically conveying program culture through a virtual format be explored and developed.

Program culture encompasses numerous features of the professional environment and may be considered within various frameworks. For example, a survey study of surgical program culture proposed establishing eight empirical features of program culture, including frequency of duty hour violations, sexual harassment, severe stress, burnout, and gender discrimination [17]. However, our fellows communicated the cultural features they valued in more positive terms, such as respectful treatment, valuing trainees as individuals and seeking their opinions, positive interactions with nursing and other staff, and genuine program diversity. Fellows also valued the ability to connect with other fellows currently in training within the program during the interview process to evaluate the degree of personal support within the program and determine shared values. Ensuring informal time for applicants to meet with current trainees is a key recommended best practice for the virtual interview format [18] for providing first-hand information on the training environment through direct interactions. We observed that having ample opportunities for candidates to interact with current trainees who were not prompted (and without faculty presence) helped candidates understand program culture.

Importantly, program cultures inherently contain some biases, and having strategies to mitigate bias that can occur during an interview process is important [14,16,19]. The Alliance for Academic Internal Medicine (AAIM) recommends that programs provide training for faculty and staff on ways to mitigate implicit bias during interviews [20]. In an effort to improve representation in the physician workforce, the ACGME requires that all programs work to recruit and retain a diverse and inclusive workforce [21]. Applicants from groups that are underrepresented in medicine may want to evaluate the diversity landscape of a program, which could be challenging in a virtual format that limits an applicant’s ability to observe the institution as a whole. Therefore, programs should implement strategies for accurately conveying the faculty, patient, and trainee populations at their institutions during virtual interviews by both providing explicit information and demonstrating it directly through the faculty and trainees who participate in the interview day [19]. Our virtual interviews were deliberately organized to include faculty who reflected the many levels of diversity in our program, and we scheduled opportunities to discuss patient populations and elements of the educational structure that address how to provide equitable care to all patients. Our fellows conveyed that diversity was important to them, and we feel that our approach helped candidates evaluate this aspect of our program’s culture.

A Best Evidence Medical Education systematic review on virtual interviewing for GME recruitment found that one weakness of virtual interviews was that they are sub-optimal for allowing programs and applicants to evaluate potential “fit” within a program [18]. Generally, the process of seeking “fit” within a program refers to an applicant’s ability to assess how well they might align with training expectations (knowledge, skills, and attitudes) as well as the program’s mission, culture, and learning environment. Thus, while applicants and organizations should consider different types of fit within a working environment (e.g., person-organization, person-job, and person-team fits) [22], determining fit for any particular program can vary depending on how “fit” is defined and viewed by all parties [23]. Notably, ensuring that “fit” does not merely imply “similarity” during the recruitment process is critical. When the concept of “fit” is not clearly defined, then the perception of candidate “fit” can promulgate unconscious bias through an overreliance on interviewer subjectivity and personal preferences. Emphasizing the complexity of program fit, one study at a university-based hospital in the United States showed that residents who had been selected through a behaviorally anchored rating scale designed to measure program fit outperformed those who were selected based on academic achievement alone [24]. For our recruiting purposes, we consider that a good fit is more than whether a candidate will get along with colleagues (person-team); rather, it reflects how well professional values align between the candidate and the training program environment. Just as it is important to provide candidates an opportunity to express what makes them uniquely qualified for training, it is equally important that programs communicate their distinctive characteristics to candidates. Overall, programs might improve the assessment of fit during virtual interviews by providing unconscious bias training to interviewers, explicitly conveying program values to applicants, asking applicants what they value, asking behavioral-based questions, planning high-quality virtual tours of the institution, and providing opportunities for spontaneous interactions with faculty and current fellows [23].

In our experience, we believe that our candidates were able to adequately assess culture during virtual interviews because we explicitly included it as a part of the interview process by preparing the faculty interviewers to clearly convey our program's values and culture and by scheduling ample time for candidates to meet virtually with current fellows. Approaches to improve virtual interactions with the current fellows might be to prepare culture-related "ice breaker" topics to discuss, which might help the individuals make a personal connection more quickly. Importantly, these interactions should be conducted within a safe space for the candidate to feel comfortable in asking candid questions; thus, individuals at the institution should conduct their discussions while in a private room with no distractions or interruptions. Providing protected time for the current fellows would help minimize work-related interruptions. And while it may be difficult for candidates to create a personal connection or assess non-verbal communication through a virtual format, preparing the interview team (both faculty and current fellows) ahead of time in practices to mitigate these challenges would be helpful, such as learning techniques that enhance engaging behavior over a virtual format. Facilities and resources are also difficult to evaluate over a virtual format, which can limit the candidate's ability to observe subtle environmental cues that might be indicative of the program's culture. Conducting in-depth virtual tours of the facilities might help a bit, but this challenge cannot be entirely eliminated. Also, because the virtual interview relies on technology, ensuring that both parties have quality computer and internet resources for a smooth connection is critical, and having a contingency plan for when technological glitches occur is important. Overall, enhanced clarity and transparency during discussions of program culture during virtual and in-person interviews benefit both applicants and programs by promoting mutual understanding and revealing areas of misalignment in this subtle but important training program feature.

Limitations

This study was performed within a single program in a large teaching hospital with a small convenience sample. Reflective interviews have the risk of recall bias, and fellows may have forgotten details about their initial recruitment-interviews, which for some had occurred almost two years prior to the study. While not intended to be reproducible, the findings may be transferable and stimulate further research. Multi-specialty and multi-center mixed methods studies addressing candidates’ perspectives on how they perceived specific domains of culture during virtual interviews would reveal how individuals in different regions and medical disciplines view training program culture. While this study included fellows who engaged in virtual and in-person formats, we note that this was not a comparative study, since qualitative studies are not aimed at generating generalizable findings. Rather, our primary interest was to improve our virtual interviewing processes through exploring how fellows who participated in virtual interviews gauged the culture of a program without being present at the institution.

## Conclusions

Overall, fellows reported that they were able to adequately evaluate our critical care training program’s culture through a virtual interview process. Whether fellows engaged with faculty and trainees virtually or in person, they were able to have meaningful conversations and make keen observations about the training environment. This suggests that the quality of interactions between applicants and interviewers may be as important, if not more important, than the practical organizational format of the interview process, whether in-person or virtual. We recommend that whether training program recruiting interviews are conducted in person or virtually, sufficient time should be provided for candidates to meaningfully engage with a representative group of faculty and current trainees, and program culture and values should be explicitly discussed. Because virtual interviews reduce the cost, environmental impact, and burden of travel inherent to in-person interviews, we endorse virtual recruiting activities that include ample effort for conveying program culture so that a broader range of candidates can participate.
